# Detection and prognostic stratification of left ventricular systolic dysfunction in left bundle branch block using an artificial intelligence–enabled electrocardiography

**DOI:** 10.1186/s44348-026-00066-9

**Published:** 2026-02-16

**Authors:** Soo Youn Lee, Ah-Hyun Yoo, Sora Kang, Jong-Hwan Jang, Yong-Yeon Jo, Jeong Min Son, Min Sung Lee, Ga In Han, Joon-myoung Kwon, Hak Seung Lee, Kyung-Hee Kim

**Affiliations:** 1 Division of Cardiology, Department of Internal Medicine, Incheon Sejong Hospital, Cardiovascular Center, Incheon, Republic of Korea; 2 Digital Healthcare Institute, Sejong Medical Research Institute, Bucheon, Republic of Korea; 3 Medical AI Co Ltd, Seoul, Republic of Korea

**Keywords:** Left ventricular systolic dysfunction, Electrocardiography, Artificial intelligence, Echocardiography, Artificial intelligence–enabled electrocardiography

## Abstract

**Background:**

Left bundle branch block (LBBB) significantly increases the risk of left ventricular systolic dysfunction (LVSD) due to cardiac dyssynchrony. Although artificial intelligence–enabled electrocardiography (AI-ECG) models show promise in detecting LVSD, their performance in LBBB patients remains underexplored. We hypothesized that an AI-ECG model clinically validated for detecting LVSD would accurately detect LVSD and predict future clinical outcomes in LBBB patients.

**Methods:**

In this retrospective multicenter study, 5,689 expert-validated LBBB ECGs collected from 2,813 patients between 2016 and 2024 were analyzed using a previously developed and validated AI-ECG model. LVSD was defined as an ejection fraction of ≤ 40%. Model performance was assessed using the area under the receiver operating characteristic curve (AUROC), the area under the precision-recall curve (AUPRC), sensitivity, and specificity. Patients were stratified into high- and low-risk groups based on a threshold that achieved 90% sensitivity. A Kaplan–Meier analysis was used to compare clinical outcomes.

**Results:**

Among the 2,813 LBBB patients (mean age, 70.7 years; male sex, 43.7%), hypertension and a history of heart failure were common. The AiTiALVSD model showed strong diagnostic performance for LVSD (AUROC, 0.930 [95% CI, 0.924–0.937]; AUPRC, 0.913 [95% CI, 0.902–0.923]; sensitivity, 0.979; specificity, 0.473). During the mean follow-up of 4.1 years, high-risk patients had significantly higher hazards than low-risk patients for all-cause mortality (adjusted hazard ratio [HR], 1.87; 95% CI, 1.53–2.28), implantable cardioverter defibrillator/cardiac resynchronization therapy implantation (adjusted HR, 15.2; 95% CI, 7.51–30.77), and cardiovascular hospitalization (adjusted HR, 1.11; 95% CI, 0.96–1.28).

**Conclusions:**

AiTiALVSD effectively detects LVSD and stratifies long-term cardiovascular risk in LBBB patients, supporting its clinical utility for early detection and patient management.

**Supplementary Information:**

The online version contains supplementary material available at 10.1186/s44348-026-00066-9.

## Background

Left bundle branch block (LBBB), characterized by QRS prolongation and electrical dyssynchrony, is closely associated with left ventricular systolic dysfunction (LVSD) due to its detrimental effects on ventricular mechanics [1–3]. Multiple studies have reported that patients with LBBB have a substantially higher than average risk of developing LVSD, with long-term follow-up data (2–7 years) showing that 20% to 40% of them experience significant declines in LV function [4–6]. This substantial risk underscores the need for effective monitoring strategies that can identify high-risk individuals early.

In clinical practice, electrocardiography (ECG) is used to identify LBBB, but this test alone cannot determine whether LVSD is present or how severe it might be. Although echocardiography is the recommended gold standard for assessing LV function in newly diagnosed LBBB, routine follow-up is often limited by cost, access, and the need for expertise [1, 7]. Even patients with initially preserved ejection fraction (EF) can later develop dysfunction, highlighting the need for practical monitoring tools [2, 8].

Recent advances in artificial intelligence (AI) have enabled the development of ECG-based models that can predict LVSD with high accuracy across diverse populations, demonstrating area under the receiver operating characteristic curve (AUROC) values that consistently exceed 0.9 [9–11]. Our AiTiALVSD model (Medical AI Co Ltd) has shown robust performance for detecting LVSD (AUROC, 0.962) in large-scale retrospective cohorts and multiple clinical settings [12–14]. However, its performance in LBBB, an electrophysiologically distinct group at high risk for LVSD, remains largely unexamined [15].

In this study, we evaluate the diagnostic performance of our AI-enabled ECG (AI-ECG) model in detecting LVSD in a cohort composed exclusively of LBBB patients. We also assess the correlation between the AI-ECG score and echocardiographic parameters, including EF, and investigate the model’s ability to predict future LV dysfunction and clinical outcomes. By focusing on this high-risk subgroup, we are gathering evidence about the diagnostic performance and potential clinical utility of using AI-ECG as a screening tool to identify LBBB patients who might benefit from early echocardiographic evaluation and timely intervention.

## Methods

### Ethics statement

The study protocol was approved by the institutional review boards of Bucheon Sejong Hospital (No. BSH 2024–09-003–005) and Incheon Sejong Hospital (No. ISH 2024–09-001). The requirement for written informed consent was waived due to the retrospective design, use of fully anonymized data, and minimal risk to participants. All procedures were performed in accordance with the principles of the Declaration of Helsinki and its later amendments.

### Study design

This retrospective multicenter study was conducted at two government-designated heart specialty hospitals in the Republic of Korea: Bucheon Sejong Hospital and Incheon Sejong Hospital. ECG data were collected at Bucheon Sejong Hospital from January 2016 to December 2024 and Incheon Sejong Hospital from January 2017 to December 2024.

### Study population and data collection

We included adult patients (aged 18 years or older) who had a 12-lead ECG showing LBBB and a paired echocardiogram performed within 14 days. LBBB labeling was performed using a two-step process. First, the automated interpretation feature of the MUSE Cardiology Information System (GE Healthcare) was used to preselect ECGs with possible LBBB from the entire hospital database. Second, all the preselected ECGs underwent manual review by an ECG specialist to confirm the diagnosis, ensuring a curated dataset of LBBB cases. The final analysis comprised 5,689 expert-validated LBBB ECGs from 2,813 patients (2016–2024).

All standard 12-lead ECGs were recorded for 10 s at a sampling rate of 500 Hz using PAGEWRITER TC30 and TC70 devices (Philips) and stored in the MUSE system. ECG features (heart rate, PR interval, QRS duration, QT interval, corrected QT interval, and P, R, and T axes) were extracted from the MUSE system. Baseline demographic and clinical data (age, sex, body mass index, systolic blood pressure, diastolic blood pressure, comorbidities [hypertension, diabetes mellitus, atrial fibrillation, ischemic heart disease, chronic kidney disease, heart failure], and echocardiographic findings, including LVEF, LV global longitudinal strain, LV end-diastolic diameter, LV end-systolic diameter, left atrial size, E/e’, E/A ratio, left atrial volume index, tricuspid regurgitation velocity, septal e’ velocity, and presence of significant mitral regurgitation, tricuspid regurgitation, or aortic stenosis) were extracted from electronic health records. In addition, clinical outcomes (all-cause mortality, cardiovascular hospitalization, and implantable cardioverter defibrillator [ICD] or cardiac resynchronization therapy [CRT] implantation) were collected through a review of electronic health records and links with national registry data, where available. There were no missing data for EF, which was the primary outcome. For other echocardiographic parameters, cases with missing values were excluded from the respective analyses, and all-available case analyses were performed. No imputation was applied.

### Outcomes

The primary outcome was the presence of LVSD, defined as an LVEF of 40% or less on echocardiography and measured using the biplane Simpson method according to the American Society of Echocardiography guidelines. The secondary outcomes were all-cause mortality, incident reduction of LVEF ≤ 40%, cardiovascular hospitalization, and the need for ICD/CRT during follow-up. Incident reduction of LVEF ≤ 40% was defined as new-onset LVSD during follow-up among patients with preserved systolic function at baseline. Patients with baseline LVEF ≤ 40% were excluded from this specific analysis. These outcomes were ascertained through a comprehensive review of electronic health records and, when available, links with national registry data to ensure completeness.

### AI model and risk stratification

All curated LBBB ECGs were analyzed using the AiTiALVSD ver. 2.00.00 AI-ECG model that we developed to detect LVSD from raw 12-lead ECG data. Our model uses a deep learning architecture that combines a convolutional neural network with transformer layers. Training was conducted using a large, multi-institutional dataset comprising 449,586 ECGs from 167,678 patients across 16 large hospitals. The algorithm processes full 10-s digital ECG waveforms without requiring manual feature engineering and generates a probability score (range, 0–100) reflecting the likelihood of LVSD for each ECG. Bucheon Sejong Hospital contributed ECG data to the original multi-institutional dataset used to develop the AiTiALVSD model. However, all ECGs and patients from Bucheon Sejong Hospital included in the present validation study were explicitly excluded from the development dataset and represent a temporally and patient-level independent cohort. Incheon Sejong Hospital did not contribute any data to the model development process. Therefore, the development and validation populations did not overlap.

The Korean Ministry of Food and Drug Safety authorized the AiTiALVSD model in 2024 as AI/machine learning (ML)-based software as a medical device, and its excellent diagnostic performance for LVSD, demonstrated in the pivotal study, was subsequently confirmed in a large-scale, retrospective analysis [14, 15]. To facilitate clinical risk stratification in this study, patients were categorized into high- and low-risk groups based on their AiTiALVSD scores using a threshold that achieved 90% sensitivity for detecting LVSD in the development dataset.

### Statistical analysis

Continuous variables are summarized as means ± standard deviations, and categorical variables are summarized as counts and percentages. The diagnostic performance of the AI-ECG model was evaluated using receiver operating characteristic curve analyses that calculated the AUROC and estimated the 95% confidence intervals (CIs). The optimal cutoff point was determined based on a threshold previously derived from the development dataset to achieve approximately 90% sensitivity for detecting LVSD. In this study, that threshold was applied to the validation cohort. Sensitivity, specificity, positive predictive value (PPV), and negative predictive value (NPV) were calculated at that predefined threshold.

For time-to-event outcomes, Kaplan–Meier curves were generated and compared with the log-rank test. Cox proportional hazards models were used to evaluate associations between AI-based LVSD risk categories and clinical endpoints, with multivariable models adjusted for age, sex, and major comorbidities. The results are expressed as hazard ratios (HRs) with 95% CIs, and proportional hazards assumptions were verified using Schoenfeld residuals. To further assess clinical applicability, model performance was evaluated using alternative EF thresholds (≤ 35% and ≤ 50%). A regression version of the AI-ECG model was also developed using transfer learning to predict continuous LVEF, and its relationship with echocardiographic LVEF was examined using scatterplots and Pearson correlation coefficients. All analyses were performed using R ver. 4.3.1 (R Foundation for Statistical Computing). A two-sided P-value of < 0.05 was considered statistically significant.

### AI explainability

To improve interpretability, gradient-based saliency mapping was applied to raw 12-lead ECGs to identify the signal regions that most influenced the LVSD predictions. Saliency maps were generated for each ECG by computing gradients of the model output with respect to input signals. Patients were stratified into high- and low-risk groups using the predefined 90% sensitivity threshold, and representative maps from each group were qualitatively compared to highlight differences in activation patterns across leads and time.

## Results

### Baseline characteristics

Our study population included 2,813 expert-validated LBBB patients (mean age, 70.7 ± 12.1 years; male sex, 43.7%) with paired echocardiography results, and 1,157 (41.1%) had LVSD. The baseline characteristics are summarized in Table 1. Compared with those without LVSD, patients with LVSD were more often male and had higher prevalences of diabetes, ischemic heart disease, chronic kidney disease, and prior heart failure, and their systolic blood pressure was lower. The model analyzed 5,689 LBBB ECGs and found significant electrophysiological differences between groups. The overall mean QRS duration was 153.4 ± 19.2 ms, with a more pronounced conduction delay and higher resting heart rates in the LVSD group. The AiTiALVSD scores also differed markedly between groups, averaging 69.4 in the LVSD group and 18.2 in the non-LVSD group.

**Table 1:** Baseline demographic, clinical, echocardiographic, and ECG characteristics of the study population

Characteristic	Total (n = 2,813)	LVSD (n = 1,157)	Non-LVSD (n = 1,656)	P-value
Demographic				
Age (yr)	70.7 ± 12.1	70.3 ± 12.0	71.0 ± 12.2	0.127
Male sex	1,230 (43.7)	642 (55.5)	588 (35.5)	< 0.001
Height (cm)	158.7 ± 10.4	160.6 ± 10.3	157.3 ± 10.3	< 0.001
Weight (kg)	61.6 ± 12.6	62.1 ± 12.8	61.3 ± 12.5	0.124
Body mass index (kg/m^2^)	24.7 ± 11.8	24.2 ± 7.2	25.1 ± 14.1	0.039
Systolic blood pressure (mmHg)	123.1 ± 20.1	118.5 ± 19.7	126.4 ± 19.7	< 0.001
Diastolic blood pressure (mmHg)	67.4 ± 12.8	66.8 ± 13.9	67.9 ± 11.9	0.062
Medical history				
Hypertension	1,488 (52.9)	562 (48.6)	926 (55.9)	0.005
Diabetes mellitus	738 (26.2)	381 (32.9)	357 (21.6)	< 0.001
Atrial fibrillation	688 (24.5)	318 (27.5)	370 (22.3)	0.045
Ischemic heart disease	1,163 (41.3)	536 (46.3)	627 (37.9)	< 0.001
Chronic kidney disease	275 (9.8)	160 (13.8)	115 (6.9)	< 0.001
Heart failure	1,831 (65.1)	959 (82.9)	872 (52.7)	< 0.001
Echocardiography				
LVEF (%)	44.0 ± 14.3	28.9 ± 7.0	54.5 ± 6.8	< 0.001
LVEDD (mm)	52.2 ± 9.6	59.3 ± 9.4	47.2 ± 5.8	< 0.001
LVESD (mm)	38.6 ± 12.0	49.1 ± 10.5	31.3 ± 6.0	< 0.001
LVGLS (%)	–12.6 ± 4.8	–8.6 ± 3.3	–14.7 ± 4.0	< 0.001
LA size (mm)	43.6 ± 14.5	44.7 ± 8.8	42.9 ± 17.4	0.001
E/E’	15.8 ± 8.3	17.3 ± 9.4	14.8 ± 7.4	< 0.001
E/A ratio	0.9 ± 0.6	1.0 ± 0.8	0.8 ± 0.5	< 0.001
LAVI (mL/m^2^)	50.6 ± 29.4	54.8 ± 33.3	47.7 ± 26.1	< 0.001
TR velocity (cm/sec)	25.8 ± 10.8	26.6 ± 11.8	25.3 ± 10.0	0.002
Septal e' velocity (cm/sec)	4.6 ± 1.7	4.1 ± 1.5	4.9 ± 1.7	< 0.001
Significant MR (%)	399 (14.2)	250 (21.6)	149 (9.0)	< 0.001
Significant TR (%)	225 (8.0)	100 (8.6)	125 (7.5)	0.893
Significant AS (%)	110 (3.9)	42 (3.6)	68 (4.1)	0.982
ECG				
No. of ECGs	5,689	2,506	3,183	
Heart rate (bpm)	75.8 ± 18.2	78.7 ± 19.4	73.5 ± 17.0	< 0.001
PR interval (msec)	188.1 ± 44.1	186.6 ± 43.6	189.3 ± 44.6	0.039
QT interval (msec)	456.7 ± 52.9	454.9 ± 53.3	458.1 ± 52.5	0.021
QRS duration (msec)	153.4 ± 19.2	158.5 ± 21.9	149.4 ± 15.5	< 0.001
Corrected QT interval (msec)	504.3 ± 40.3	511.4 ± 41.5	498.7 ± 38.4	< 0.001
P axis	44.7 ± 43.3	47.7 ± 43.9	42.3 ± 42.7	< 0.001
R axis	–8.3 ± 34.6	–10.7 ± 36.2	–6.4 ± 33.2	< 0.001
T axis	132.9 ± 61.5	140.6 ± 67.0	126.9 ± 56.1	< 0.001
AI-ECG score (AiTiALVSD)	40.7 ± 33.5	69.4 ± 24.6	18.2 ± 19.3	< 0.001

Values are presented as mean ± standard deviation or number (%), unless otherwise indicated. Baseline characteristics of the 2,813 patients with left bundle branch block included in the study

AI-ECG, artificial intelligence–enabled electrocardiography; AS, aortic stenosis; bpm, beats per minute; ECG, electrocardiography; LA, left atrial; LAVI, left atrial volume index; LVEDD, left ventricular end-diastolic diameter; LVEF, left ventricular ejection fraction; LVESD, left ventricular end-systolic diameter; LVGLS, left ventricular global longitudinal strain; LVSD, left ventricular systolic dysfunction; MR, mitral regurgitation; TR, tricuspid regurgitation

### Diagnostic performance of AiTiALVSD

**Fig. 1:**
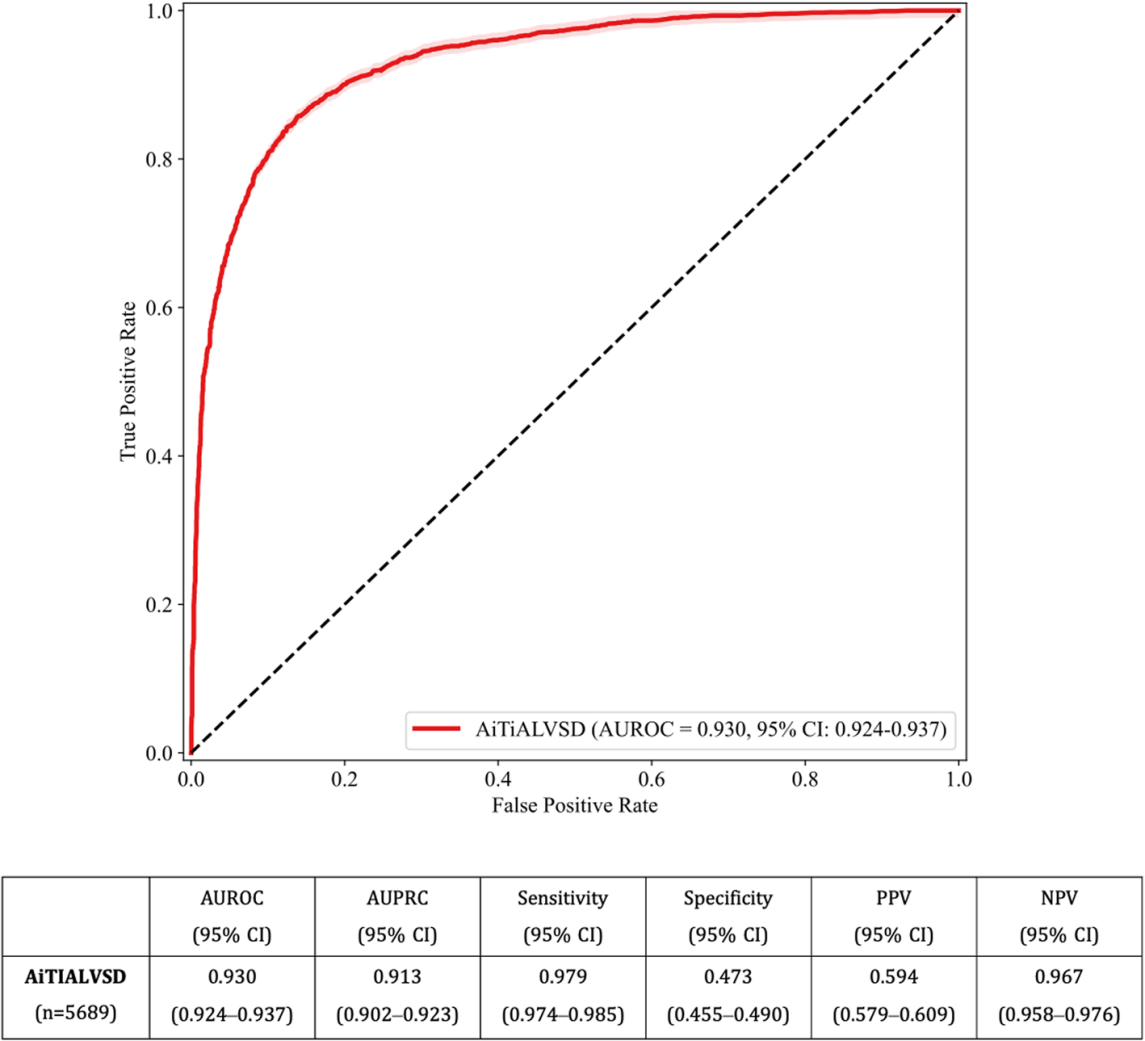
Diagnostic performance of the AiTiALVSD model for detecting left ventricular systolic dysfunction (LVSD) in left bundle branch block (LBBB) patients. The receiver operating characteristic curve illustrates the diagnostic performance of the AiTiALVSD model for identifying LVSD in the LBBB cohort, with an area under the receiver operating characteristic curve (AUROC) of 0.930 (95% confidence interval [CI], 0.924–0.937). The red line represents the model’s performance, and the dashed diagonal indicates the line of no discrimination. The model achieved high AUROC (0.930), sensitivity (97.9%), and negative predictive value (NPV; 96.7%) at the predefined threshold. AUPRC, area under the precision-recall curve

The AiTiALVSD model demonstrated excellent diagnostic accuracy for LVSD in LBBB patients, achieving an AUROC of 0.930 (95% CI, 0.924–0.937) and area under the precision-recall curve (AUPRC) of 0.913 (95% CI, 0.902–0.923). Sensitivity was 0.979 (95% CI, 0.974–0.985), specificity 0.473 (95% CI, 0.455–0.490), PPV 0.594 (95% CI, 0.579–0.609), and NPV 0.967 (95% CI, 0.958–0.976) (Fig. 1).

Performance remained consistently strong across clinically relevant LVEF thresholds. Using an LVEF cutoff of 35%, the model achieved an AUROC of 0.927 (95% CI, 0.921–0.934), with 99.0% sensitivity, 42.7% specificity, and an NPV of 98.7%, effectively ruling out severe systolic dysfunction. (Table 2). When a 50% cutoff was applied, the model yielded an AUROC of 0.907 (95% CI, 0.899–0.914), with 93.8% sensitivity and 58.3% specificity, maintaining high diagnostic accuracy across varying severities of LV dysfunction.

**Table 2:** Diagnostic performance of the AiTiALVSD model for detecting LVSD at different LVEF thresholds in LBBB patients

	AUROC (95% CI)	AUPRC (95% CI)	Sensitivity (95% CI)	Specificity (95% CI)	PPV (95% CI)	NPV (95% CI)
LVEF ≤ 35%	0.927 (0.921–0.934)	0.875 (0.861–0.889)	0.990 (0.986–0.994)	0.427 (0.410–0.444)	0.501 (0.486–0.517)	0.987 (0.981–0.992)
LVEF ≤ 50%	0.907 (0.899–0.914)	0.937 (0.931–0.944)	0.938 (0.929–0.946)	0.583 (0.562–0.604)	0.767 (0.755–0.779)	0.865 (0.847–0.881)

Performance metrics (AUROC, AUPRC, sensitivity, specificity, PPV, and NPV with 95% CIs) are shown for alternative thresholds of LVEF ≤ 35% and ≤ 50%. The model achieved high sensitivity and NPV across all thresholds, with AUROC values of 0.927 (LVEF ≤ 35%) and 0.907 (LVEF ≤ 50%)

AUPRC, area under the precision-recall curve; AUROC, area under the receiver operating characteristic curve; CI, confidence interval; LBBB, left bundle branch block; LVEF, left ventricular ejection fraction; LVEF, left ventricular ejection fraction; LVSD, left ventricular systolic dysfunction; NPV, negative predictive value; PPV, positive predictive value

To further examine the stability of model performance across a wider spectrum of thresholds, we performed sensitivity analyses using cutoff values from 20 to 90 (Table S1). As the threshold increased, sensitivity decreased and specificity and PPV improved, illustrating a trade-off and the flexibility to tailor the threshold according to clinical priorities. Overall, the model demonstrated consistent, high-level performance across multiple LVEF thresholds and decision cutoffs, supporting its applicability in diverse clinical scenarios.

### Correlation between AiTiALVSD score and LVEF

**Fig. 2:**
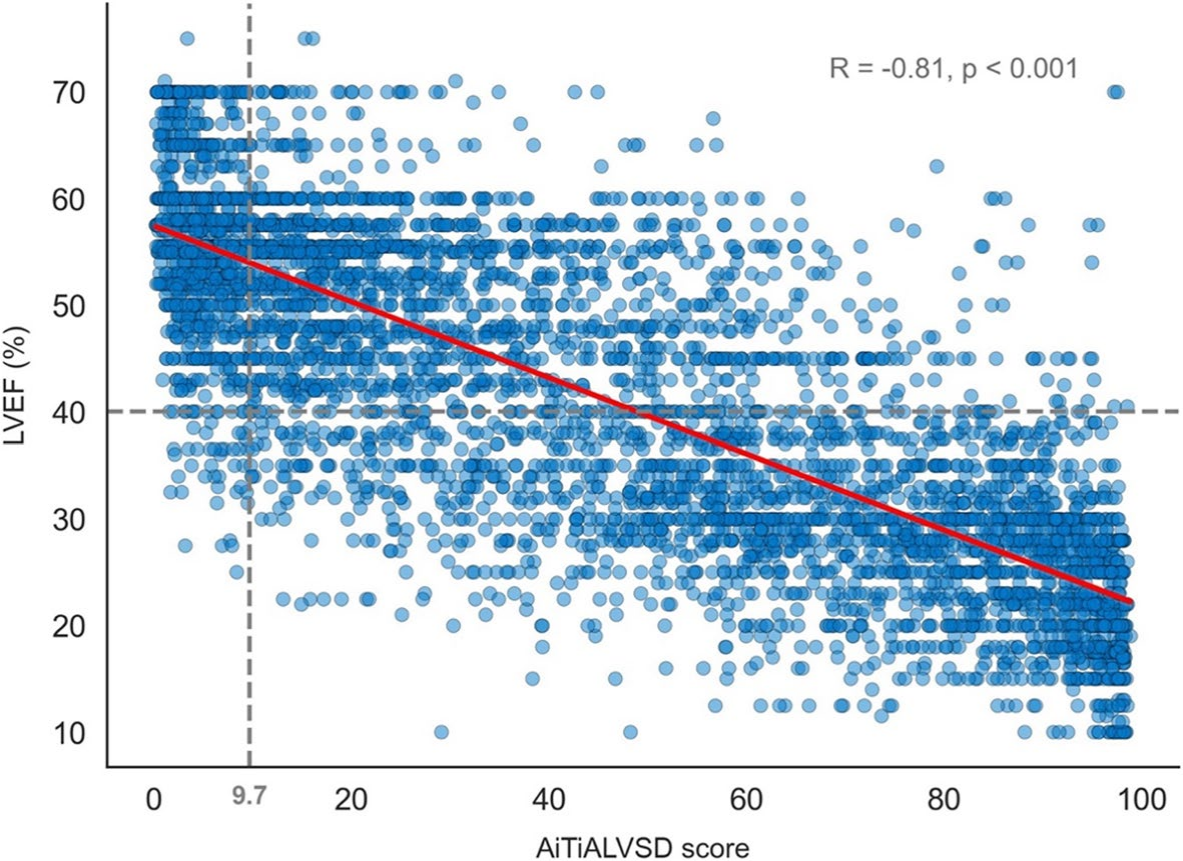
Correlation between AiTiALVSD scores and left ventricular ejection fraction (LVEF) in left bundle branch block patients. Scatterplot showing a strong inverse correlation between the AiTiALVSD score and LVEF (R = –0.81, P < 0.001). The vertical dashed line marks the AiTiALVSD threshold (9.7), and the horizontal dashed line indicates the left ventricular systolic dysfunction (LVSD) cutoff

The AiTiALVSD score demonstrated a strong inverse correlation with echocardiographic LVEF (R = –0.81, P < 0.001). As shown in Fig. 2, higher scores were consistently associated with lower LVEF across the full range of values, with patients who had LVSD clustering in the higher score range and those with preserved EF clustering in the lower range, supporting the model’s ability to reflect the severity of LVSD.

### Subgroup analyses

The diagnostic performance of the AiTiALVSD model remained robust across clinically relevant subgroups (Fig. S1, Table S2). AUROC values consistently exceeded 0.91 across all subgroups. The model demonstrated consistently high sensitivity (> 96%) across all subgroups, with particularly strong performance in diabetes patients (sensitivity, 99.6%; NPV, 99.0%). Performance remained stable regardless of age, sex, comorbidities, and QRS duration, supporting the model's broad clinical applicability in LBBB patients with diverse risk profiles.

### Prognostic value for long-term clinical outcomes

During a mean follow-up of 4.1 years, high-risk patients identified by the AI-ECG model exhibited significantly higher hazards for all-cause mortality (adjusted HR, 1.87; 95% CI, 1.53–2.28), incident reduction of LVEF ≤ 40% (adjusted HR, 12.05; 95% CI, 7.60–19.10), ICD/CRT implantation (adjusted HR, 15.2; 95% CI, 7.51–30.77), and cardiovascular hospitalization (adjusted HR, 1.11; 95% CI, 0.96–1.28), compared with the low-risk group (all P < 0.001; except cardiovascular hospitalization, P = 0.150).

**Fig. 3:**
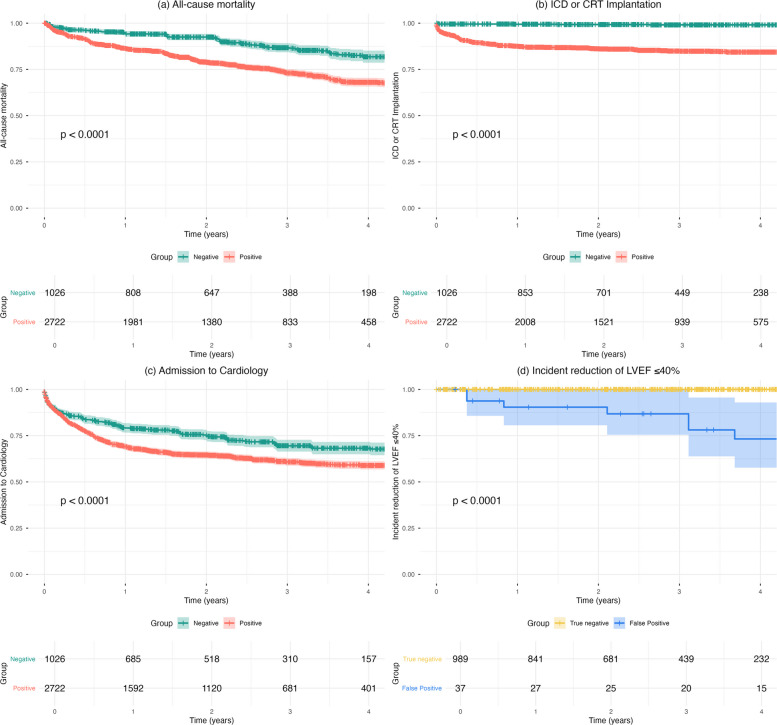
Kaplan–Meier curves for clinical outcomes according to artificial intelligence–enabled electrocardiography risk stratification in left bundle branch block patients. Kaplan–Meier survival curves comparing the high- and low-risk groups (as determined by the AiTiALVSD score) for (**A**) all-cause mortality, (**B**) implantable cardioverter defibrillator (ICD) or cardiac resynchronization therapy (CRT) implantation, and (**C**) admission to cardiology. **D** Kaplan–Meier curve for incident left ventricular systolic dysfunction (new-onset left ventricular ejection fraction [LVEF] ≤ 40%) among patients with preserved systolic function at baseline (LVEF > 40%). Patients with baseline LVEF ≤ 40% were excluded from this analysis. High-risk patients exhibited significantly higher rates of adverse outcomes across all endpoints, with clear separation between risk strata (log-rank P < 0.001 for all comparisons)

Kaplan–Meier analyses demonstrated that patients classified as high risk by the AI-ECG model had significantly higher incidences of all-cause mortality, ICD/CRT implantation, and admission to cardiology, compared with the low-risk group, with clear separation of the survival curves throughout the follow-up period (log-rank P < 0.001 for all) (Fig. 3A–C). In addition, among patients with preserved left ventricular systolic function at baseline (LVEF > 40%), those categorized as high risk by the AI-ECG model exhibited a significantly higher incidence of new-onset LVSD (LVEF ≤ 40%) during follow-up, compared with the low-risk group (Fig. 3D).

### Saliency map analysis: distinct activation in high-risk LBBB patients

**Fig. 4:**
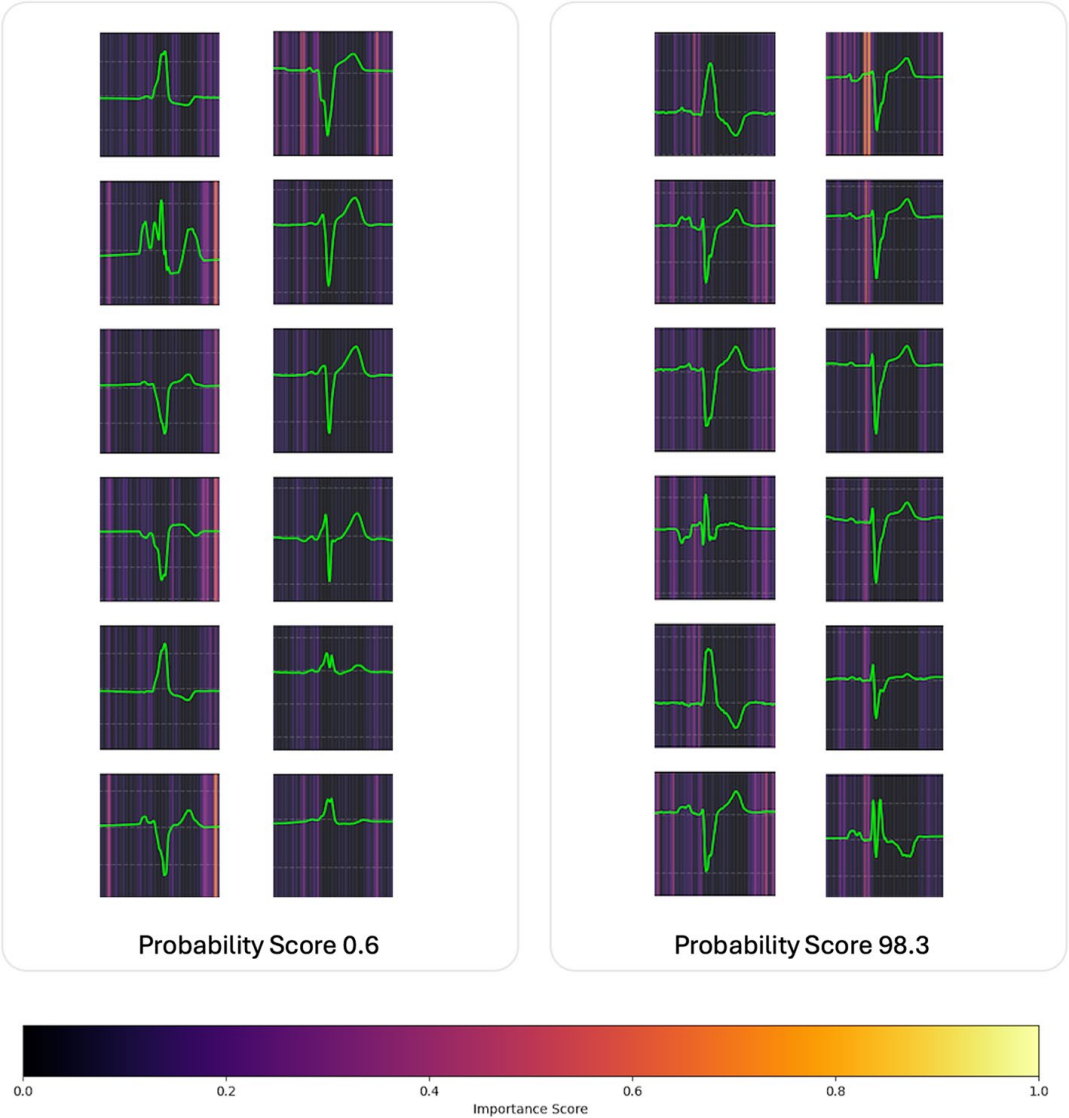
Saliency map comparison between the low- and high-risk groups. Saliency maps highlight differences in model attention: high-risk left bundle branch block patients show strong activation in the V1 lead at the PR interval, whereas low-risk patients lack clear focal activation

The saliency map analysis of 12-lead ECGs revealed distinct activation patterns between the low- and high-risk groups classified by the AI-ECG model (Fig. 4). In high-risk patients, there was pronounced saliency within the V1 lead, particularly at the PR interval segment, indicating that the model consistently focused on this region when predicting LVSD. In contrast, the saliency maps for the low-risk group displayed more diffuse or less intense activation across leads and timepoints, without a dominant focus.

## Discussion

In this multicenter cohort of patients with LBBB, we demonstrated that an AI-enabled ECG model can not only detect LVSD with high accuracy but also stratify long-term clinical risk, despite the electrophysiologic complexity that often complicates interpretation in this population. Overall, our findings suggest that AI-ECG can help bridge the gap between simple but insensitive approaches, such as ECG morphology criteria, and resource-intensive imaging performed with echocardiography by providing a continuous, scalable risk signal that is both diagnostically and prognostically meaningful in LBBB.

We highlight three key implications. First, despite concerns that LBBB might degrade AI-ECG performance, the AiTiALVSD model retained excellent diagnostic accuracy for LVSD detection. Second, the AI-derived risk score showed strong correlation with LVEF and independent associations with major adverse outcomes, supporting its role as a dynamic marker—and potential digital biomarker—linking electrical signals to structural and clinical risk. Third, because the model can be integrated directly into routine ECG workflows and is technically feasible for real-world use, our findings advance AI-ECG for LVSD in LBBB from a theoretical possibility to a practical clinical tool.

### High diagnostic performance of AI-ECG screening in LBBB patients

LBBB has traditionally been regarded as an ECG blind spot for assessing LVSD. The wide QRS complexes, secondary ST-T abnormalities, and heterogeneous conduction patterns make interpreting conventional ECG indicators of systolic dysfunction difficult, and prior studies have suggested that AI-ECG algorithms might also perform suboptimally in those conditions [16, 17]. In this context, the AUROC of 0.930 observed in our LBBB-only cohort is notable. This finding indicates that, despite substantial conduction abnormalities, the AI model can still extract latent patterns that accurately capture systolic impairment, with performance comparable to that reported in broader, non-LBBB populations [11]. The high NPV of 96.7% further demonstrates the model’s ability to reliably rule out LVSD in patients with LBBB, supporting its use as a safe and efficient screening tool to identify individuals who do not require further echocardiographic evaluation.

The very high sensitivity and NPV at the prespecified threshold are particularly relevant for clinical application. In many health systems, echocardiography is a significant bottleneck, and clinicians must determine which LBBB patients need urgent imaging and which can be safely deferred. Our findings suggest that a low AiTiALVSD score can support a rule-out strategy by identifying low-score patients in whom immediate imaging is unlikely to reveal LVSD and high-scores patients who warrant prompt echocardiographic assessment [18].

Importantly, the model also maintained strong performance across alternative LVEF thresholds such as ≤ 35% and ≤ 50%, which align more directly with therapeutic decisions (e.g., ICD/CRT eligibility) and broad gradations of systolic impairment. This robustness across clinically meaningful cut points suggests that AiTiALVSD is not narrowly tuned to a single definition of LVSD but instead captures a continuum of ventricular dysfunction. Moreover, the sensitivity–specificity trade-offs observed across different decision thresholds indicate that clinicians and health systems can tailor cutoff values depending on whether the priority is maximizing case detection (e.g., screening or case-finding programs) or minimizing false positives in settings where echocardiography capacity is limited [19].

### Prognostic value and clinical utility of AI-ECG scores

The strong inverse correlation between the AiTiALVSD score and echocardiographic LVEF has two important implications. First, it supports the construct validity of the AI score as a quantitative indicator of systolic function rather than a simple binary classifier. Second, it suggests that the electrical features extracted by the model are closely linked to underlying mechanical performance, despite the conduction delay and dyssynchrony inherent in LBBB.

However, the prognostic analyses show that the AI-ECG signal reflects more than just a surrogate for LVEF. Even after adjusting for baseline characteristics, patients classified as high risk by their AiTiALVSD scores had significantly higher hazards for all-cause mortality, new-onset reduction in LVEF, ICD/CRT implantation, and cardiovascular hospitalization during follow-up. Importantly, the association with new-onset LVSD remained significant when the analysis was restricted to patients with preserved systolic function at baseline, underscoring the ability of the AI-ECG model to identify individuals at risk for subsequent deterioration in ventricular function. This aligns with findings from other populations in which AI-ECG scores predicted adverse events independently of imaging-based measures and traditional risk factors [20, 21].

Clinically, these findings point to two practical applications. First, when LBBB is newly identified, the AiTiALVSD score can help identify patients who already have LVSD or are at increased risk, guiding timely echocardiography, closer surveillance, and earlier initiation of appropriate therapy. Second, because ECGs are obtained repeatedly in routine care, the AI-ECG score could function as a digital biomarker for longitudinal monitoring; a gradual rise in previously low-risk patients could signal emerging dysfunction before an overt decline in LVEF. Although we did not quantify such trajectory-based utility in this study, the strong cross-sectional and prognostic associations we found provide a clear rationale for future research evaluating serial AI-ECG changes.

### Explainable AI–driven interpretive insights

The saliency map analysis offers preliminary insight into the electrical features the model might be using when predicting LVSD in patients with LBBB. The consistent prominence of the V1 lead, particularly around the PR-interval region, in high-risk patients suggests that atrioventricular conduction patterns and early septal activation might carry physiologic signals linked to downstream ventricular dysfunction [22]. This is mechanistically plausible because delayed left ventricular activation in LBBB produces mechanical dyssynchrony and associated alterations in septal motion, QRS onset, and early repolarization that reflect the cumulative effects of conduction disease and structural remodeling. The model might therefore be integrating subtle timing- and morphology-based cues in V1 and adjacent leads that are difficult for human readers to discern. In contrast, the more diffuse and lower-intensity saliency patterns seen in low-risk patients indicate that the model’s predictions are not driven simply by the presence of LBBB but rather by specific electrophysiologic configurations embedded within it.

Although the saliency analysis does not provide a complete mechanistic explanation and should be interpreted with caution, these findings highlight clear directions for future work, such as linking AI-derived activation patterns to quantitative measures of electrical dyssynchrony, myocardial strain abnormalities, or response to CRT.

### Implementation, health system effects, and the role of regulation

A key strength of this study is that it evaluates an algorithm that has already undergone regulatory review and received authorization from the Korean Ministry of Food and Drug Safety as AI/ML-based software as a medical device. This shifts the discussion from asking whether the approach could work to determining how it can best be implemented in clinical practice. Our findings, together with an increasing body of pragmatic studies on AI-ECG deployment, support an implementation model in which LVSD risk scores are generated automatically at the time of routine ECG acquisition and presented to clinicians within their existing workflow [23].

Economic considerations are equally important. Prior cost-effectiveness analyses have shown that AI-ECG–guided screening for reduced LVEF can be financially advantageous because its high NPV enables safe reduction of unnecessary echocardiograms while still identifying high-risk patients who require further testing [19, 20, 24]. Our results extend this concept to the LBBB population, precisely the group in which clinicians often struggle between performing echocardiography for all patients versus adopting a watch-and-wait approach. By enabling a more targeted, data-driven strategy, AiTiALVSD has the potential to improve resource allocation, particularly in settings where imaging capacity is limited.

### Limitations and future directions

Several limitations should be acknowledged. First, this study has a retrospective design and includes only patients who underwent both LBBB-confirming ECG and echocardiography, introducing selection bias. Asymptomatic individuals with LBBB who never received imaging are likely underrepresented, limiting generalizability to the broader LBBB population. As a result, the observed prevalence of LVSD and heart failure in this cohort was substantially higher than would be expected in an unselected LBBB population, and that might have inflated the absolute risk estimates and PPV. Therefore, the prognostic and diagnostic performance reported in this study should be interpreted in the context of a clinically enriched population, rather than a population-based screening setting. Future prospective studies are warranted to evaluate model performance in incidentally detected LBBB populations in which echocardiography is not routinely performed.

Second, despite the addition of a saliency map analysis, the model remains a deep-learning black box from a clinical perspective. Future work integrating explainable AI techniques with mechanistic imaging markers, such as myocardial strain or electrical and mechanical dyssynchrony indices, might help bridge that gap.

Third, the study population was derived from two Korean medical centers. Although the pathophysiology of LBBB is not ethnicity-specific, validation in geographically and ethnically diverse cohorts is essential. Fourth, although the mean follow-up duration of 4.1 years is considerable, it might underestimate very long-term risks and does not fully account for treatment changes over time, such as initiation of CRT or optimization of heart failure therapy, that could influence both AI-ECG scores and clinical outcomes. Fourth, we did not perform an economic evaluation, so that is an important area for future research.

## Conclusions

In this large multicenter study, the AiTiALVSD AI-ECG model demonstrated excellent diagnostic and prognostic performance for LVSD in patients with LBBB. These findings support its integration into routine clinical practice to enable early detection and risk stratification without immediate reliance on echocardiography. Moreover, AI-ECG technology can be readily scaled across diverse healthcare settings, including resource-limited environments, and holds promise as a future digital biomarker that can support proactive intervention and longitudinal management.

## Supplementary Information

Additional file 1: Fig. S1. Subgroup analysis of diagnostic performance for LVSD detection in LBBB patients. Table S1. Sensitivity analysis of the AiTiALVSD model at representative cutoff values. Table S2. Subgroup analysis of diagnostic performance for LVSD detection in LBBB patients.

## Data Availability

No datasets were generated or analysed during the current study.

## Abbreviations

AI: Artificial intelligence
AI-ECG: Artificial intelligence–enabled electrocardiography
AUPRC: Area under the precision-recall curve
AUROC: Area under the receiver operating characteristic curve
CI: Confidence interval
CRT: Cardiac resynchronization therapy
ECG: Electrocardiography
EF: Ejection fraction
HR: Hazard ratio
ICD: Implantable cardioverter defibrillator
LBBB: Left bundle branch block
LV: Left ventricular
LVEF: Left ventricular ejection fraction
LVSD: Left ventricular systolic dysfunction
ML: Machine learning
NPV: Negative predictive value
PPV: Positive predictive value
