# Supplementary material for: Detection and prognostic stratification of left ventricular systolic dysfunction in left bundle branch block using an artificial intelligence–enabled electrocardiography
Source: J Cardiovasc Imaging. 2026 Feb 16;34:4. doi: 10.1186/s44348-026-00066-9 (PMC12914959; doi:10.1186/s44348-026-00066-9)
Supplement: Supplementary file 1 — Additional file 1: Fig. S1. Subgroup analysis of diagnostic performance for LVSD detection in LBBB patients. Table S1. Sensitivity analysis of the AiTiALVSD model at representative cutoff values. Table S2. Subgroup analysis of diagnostic performance for LVSD detection in LBBB patients. [file 44348_2026_66_MOESM1_ESM.docx]

**Supplementary Figure 1.** Subgroup Analysis of Diagnostic Performance for LVSD Detection in LBBB Patients.


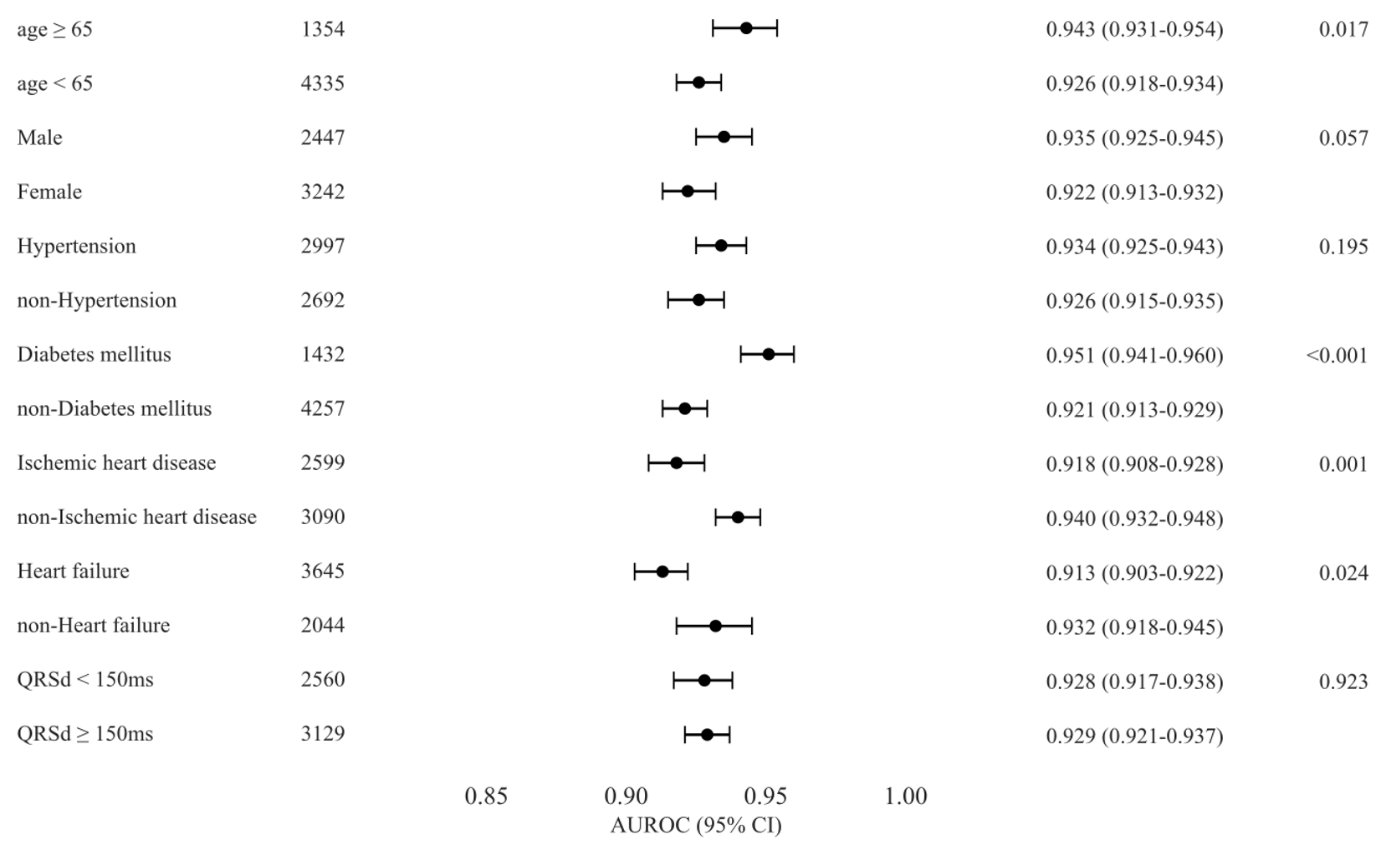


Diagnostic performance metrics across clinically relevant subgroups demonstrate consistent high performance with AUROC >0.91 in all categories.

LVSD, Left Ventricular Systolic Dysfunction; LBBB, Left Bundle Branch Block; AUROC, Area Under the Receiver Operating Characteristic curve.

**Supplementary Table 1.** Sensitivity analysis of the AiTiALVSD Model at Representative Cutoff Values.

| **Cutoff** | **Sensitivity** | **Specificity** | **PPV** | **NPV** |
| --- | --- | --- | --- | --- |
| 20 | 0.949 | 0.679 | 0.699 | 0.944 |
| 30 | 0.907 | 0.788 | 0.771 | 0.915 |
| 40 | 0.856 | 0.862 | 0.830 | 0.884 |
| 50 | 0.791 | 0.909 | 0.873 | 0.846 |
| 60 | 0.680 | 0.952 | 0.918 | 0.791 |
| 70 | 0.569 | 0.975 | 0.948 | 0.742 |
| 80 | 0.445 | 0.987 | 0.965 | 0.693 |
| 90 | 0.263 | 0.994 | 0.973 | 0.632 |

Sensitivity, specificity, positive predictive value (PPV), and negative predictive value (NPV) are shown for each cutoff.

PPV, positive predictive value; NPV, negative predictive value.

**Supplementary Table 2.** Subgroup Analysis of Diagnostic Performance for LVSD Detection in LBBB Patients

| **AiTiALVSD** | **AUROC** | **AUPRC** | | **Sensitivity** | | **Specificity** | | **PPV** | | **NPV** | |
| --- | --- | --- | --- | --- | --- | --- | --- | --- | --- | --- | --- |
|  | **(95% CI)** | | **(95% CI)** | | **(95% CI)** | | **(95% CI)** | | **(95% CI)** | | **(95% CI)** |
| Age <65  (n=1354) | 0.943 | | 0.93 | | 0.993 | | 0.477 | | 0.591 | | 0.989 |
|  | (0.931–0.954) | | (0.913–0.945) | | (0.986–0.998) | | (0.442–0.511) | | (0.563–0.619) | | (0.977–0.997) |
| Age ≥65  (n=4335) | 0.926 | | 0.908 | | 0.975 | | 0.471 | | 0.595 | | 0.959 |
|  | (0.918–0.934) | | (0.893–0.921) | | (0.967–0.982) | | (0.453–0.491) | | (0.578–0.611) | | (0.948–0.971) |
| Male  (n=2447) | 0.935 | | 0.94 | | 0.982 | | 0.422 | | 0.663 | | 0.954 |
|  | (0.925–0.945) | | (0.927–0.953) | | (0.975–0.989) | | (0.393–0.451) | | (0.641–0.683) | | (0.935–0.971) |
| Female  (n=3242) | 0.922 | | 0.875 | | 0.976 | | 0.5 | | 0.532 | | 0.972 |
|  | (0.913–0.932) | | (0.858–0.893) | | (0.967–0.984) | | (0.477–0.519) | | (0.511–0.552) | | (0.962–0.981) |
| HTN  (n=2997) | 0.934 | | 0.921 | | 0.979 | | 0.478 | | 0.576 | | 0.968 |
|  | (0.925–0.943) | | (0.909–0.932) | | (0.970–0.986) | | (0.456–0.501) | | (0.556–0.599) | | (0.956–0.980) |
| Non-HTN  (n=2692) | 0.926 | | 0.906 | | 0.98 | | 0.467 | | 0.613 | | 0.964 |
|  | (0.915–0.935) | | (0.887–0.923) | | (0.972–0.987) | | (0.442–0.494) | | (0.592–0.634) | | (0.950–0.978) |
| DM  (n=1432) | 0.951 | | 0.958 | | 0.996 | | 0.438 | | 0.682 | | 0.990 |
|  | (0.941–0.960) | | (0.947–0.967) | | (0.991–1.000) | | (0.402–0.473) | | (0.655–0.708) | | (0.976–1.000) |
| Non-DM  (4257) | 0.921 | | 0.89 | | 0.972 | | 0.481 | | 0.56 | | 0.961 |
|  | (0.913–0.929) | | (0.875–0.904) | | (0.964–0.979) | | (0.463–0.501) | | (0.541–0.579) | | (0.950–0.972) |
| IHD (n=2599) | 0.918 | | 0.916 | | 0.973 | | 0.411 | | 0.604 | | 0.942 |
|  | (0.908–0.928) | | (0.903–0.928) | | (0.964–0.982) | | (0.385–0.436) | | (0.582–0.625) | | (0.924–0.961) |
| Non-IHD (n=3090) | 0.94 | | 0.911 | | 0.986 | | 0.518 | | 0.584 | | 0.981 |
|  | (0.932–0.948) | | (0.893–0.925) | | (0.979–0.992) | | (0.496–0.541) | | (0.563–0.605) | | (0.973–0.989) |
| HF (n=3645) | 0.913 | | 0.928 | | 0.983 | | 0.386 | | 0.684 | | 0.943 |
|  | (0.903–0.922) | | (0.916–0.939) | | (0.977–0.988) | | (0.362–0.409) | | (0.667–0.699) | | (0.925–0.960) |
| Non-HF (n=2044) | 0.932 | | 0.826 | | 0.961 | | 0.554 | | 0.351 | | 0.983 |
|  | (0.918–0.945) | | (0.790–0.855) | | (0.941–0.978) | | (0.531–0.577) | | (0.323–0.378) | | (0.974–0.990) |
| QRSd <150ms  (n=2560) | 0.928 | | 0.878 | | 0.967 | | 0.547 | | 0.537 | | 0.968 |
|  | (0.917–0.938) | | (0.857–0.897) | | (0.955–0.978) | | (0.522–0.572) | | (0.512–0.564) | | (0.956–0.979) |
| QRSd ≥150ms  (n=3129) | 0.929 | | 0.931 | | 0.986 | | 0.392 | | 0.631 | | 0.965 |
|  | (0.921–0.937) | | (0.919–0.943) | | (0.980–0.992) | | (0.367–0.418) | | (0.612–0.649) | | (0.949–0.979) |

PPV, positive predictive value; NPV, negative predictive value; HGN, hypertension; DM, diabetes mellitus; IHD, ischemic heart disease; HF, heart failure.
